# Pro-resolving lipid mediator ameliorates obesity induced osteoarthritis by regulating synovial macrophage polarisation

**DOI:** 10.1038/s41598-018-36909-9

**Published:** 2019-01-23

**Authors:** Antonia Rujia Sun, Xiaoxin Wu, Bohao Liu, Yang Chen, Charles W. Armitage, Avinash Kollipara, Ross Crawford, Kenneth W. Beagley, Xinzhan Mao, Yin Xiao, Indira Prasadam

**Affiliations:** 1 Institute of Health and Biomedical Innovation, Faculty of Science and Engineering, Queensland University of Technology, Brisbane, 4059 Australia; 2 The Prince Charles Hospital, Orthopedic Department, Brisbane, Australia; 3 Department of Orthopaedic Surgery, Second Xiangya Hospital, Central South University, Changsha, China; 4 Institute of Health and Biomedical Innovation, Faculty of Health, School of Biomedical Sciences, Queensland University of Technology, Brisbane, Australia; 5 Department of Pediatrics, School of Medicine, The University of North Carolina at Chapel Hill, Chapel Hill, USA; 6 Australia–China Centre for Tissue Engineering and Regenerative Medicine, Queensland University of Technology, Brisbane, Queensland Australia

## Abstract

Non-resolved persistent macrophage-mediated synovial inflammation is considered as one of the main drivers of both the establishment and progression of obesity-associated osteoarthritis (OA). Herein, we used clodronate-loaded liposomes (CL) to locally deplete macrophages in the synovial joints to examine the role of macrophages in the progression of obesity-induced OA. Furthermore, resolvin D1 (RvD1), a unique family of pro-resolving lipid mediator derived from the omega-3 polyunsaturated fatty acid, have shown marked potency in changing the pro-inflammatory behaviour of the macrophages. We sought to determine whether RvD1 administration ameliorates obesity-induced OA by resolving macrophage-mediated synovitis. Therapeutic properties of RvD1 and macrophage depletion (CL) were tested for its ability to slow post-traumatic OA (PTOA) in obese mice models. PTOA was induced in C57Bl/6 mice fed with high-fat diet (HFD) by surgically destabilising the meniscus. Firstly, CL treatment showed beneficial effects in reducing synovitis and cartilage destruction in obese mice with PTOA. *In vitro* treatment with RvD1 decreased the levels of pro-inflammatory markers in CD14+ human macrophages. Furthermore, intra-articular treatment with RvD1 diminishes the progression of OA in the knee joint from mice as follows: (a) decreases macrophages infiltration in synovium, (b) reduces the number of pro-inflammatory macrophages in synovium and (c) improves the severity of synovitis and cartilage degradation. Thus, our results provide new evidence for the potential targeting of macrophages in the treatment of obesity-induced OA.

## Introduction

Metabolic osteoarthritis (OA) is a newly defined phenotype of OA, which is associated with metabolic syndrome (MetS) and obesity^1^. The main pathophysiologic mechanism underlying obesity-associated osteoarthritis is perpetual inflammation, resulting in cartilage loss, osteophyte formation and synovitis that lead to the development of OA^2^. We recently demonstrated that rats fed with a high-carbohydrate high-fat diet (HCHF) spontaneously developed OA and macrophage infiltration in the joint synovium compared to control diet fed mice. In addition, the infiltrated macrophages showed a pro-inflammatory M1 phenotype in synovial tissue of knee joints^3^. These data suggest that novel therapies that target macrophage polarization may mitigate the development of obesity-induced OA.

Resolvin D1 (RvD1), a pro-resolving lipid mediator, is derived from omega-3 docosahexaenoic acid during the resolution phase of inflammation, and displays potent anti-inflammatory and pro-resolving characteristics^4,5^. Resolvins, which are produced upon interactions with neutrophils, platelets and macrophages in inflamed tissues, have been shown to be potent mediators of switching macrophages from a pro-inflammatory state (M1) to anti-inflammatory (M2) when tested in inflammatory diseases *in vivo* or *in vitro*^5–7^. Considering the important role of macrophage polarisation in obesity-induced OA, we tested the hypothesis that RvD1 may mitigate obesity-induced OA progression by changing pro-inflammatory behaviour of the macrophages.

In this study, we examine the role of macrophages in the progression of obesity-induced OA. We used clodronate-loaded liposomes to locally deplete macrophages in the synovial joints to examine the role of macrophages in the progression of obesity-associated osteoarthritis. Furthermore, we tested if resolution of inflammation using the RvD1 would mitigate OA in mice models of obesity and injury (Destabilization of medial meniscus (DMM) model).

## Results

### High fat diet promotes weight gain and altered metabolic parameter

**Figure 1:**
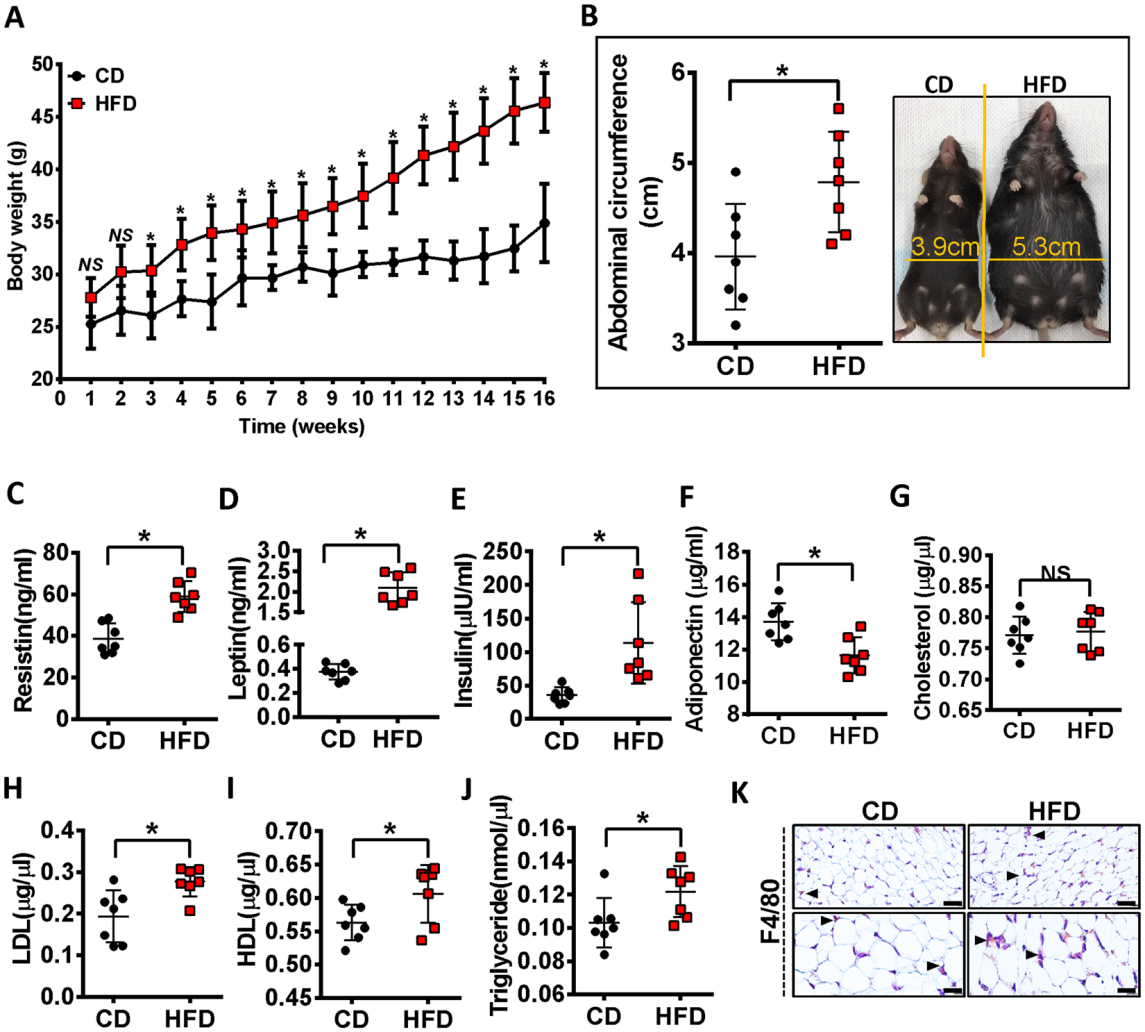
High-fat diet promotes weight gain and altered metabolic parameters. (**A**) Body weight of CD or HFD mice were monitored over 16 weeks. (**B**) Ventral view of the mice showing the changes in the total abdominal length caused by the two diets after 16 weeks. (**C**–**J**) Effect of HFD on metabolic parameters. Measurement of serum resistin (**C**), leptin (**D**), insulin (**E**), adiponectin (**F**), total cholesterol (**G**), LDL (**H**), HDL (**I**) and triglyceride (**J**). (**K**) Immunostained section of the infrapatellar fat pad (IFP) of mice fed a control or HFD diet. Bar = 100 µm. Graphs represent mean ± SD (n = 7). *p < 0.05. CD, control diet-fed mice; HFD, high fat diet-fed mice.

After 16 weeks of the HFD diet, mice showed a significant increase in body weight compared to mice fed the control diet (CD) with CD mice weighing 33.49 ± 2.28 g compared to HFD mice at 44.39 ± 2.82 g (*p* < 0.05) (Fig. 1A). In line with body weight, HFD-fed mice had an increased abdominal circumference (Fig. 1B). HFD-fed mice showed increased serum resistin, leptin, insulin levels and decreased serum adiponectin level compared to CD-fed mice (Fig. 1C–F). Furthermore, as shown in Fig. 1G–J, total cholesterol levels at 16 wk were no different in HFD-fed mice compared with CD-fed mice, but low-density lipoprotein (LDL), high-density lipoprotein (HDL) and triglyceride were markedly higher in HFD-fed mice. We next compared the effect of HFD feeding on macrophage infiltration in the adipose tissue of the infrapatellar fat pad (IFP), as determined with F4/80 staining (Fig. 1K). The IFP from HFD fed mice had an increased number of F4/80^+^ cells compared to the control animals, however, macrophage crown-like structures, another characteristic of adipose tissue inflammation^8^, were not observed in the IFP. Overall these data demonstrate that a HFD leads to factors indicative of metabolic alteration.

### High fat diet induces OA-like changes and accelerates surgically-induced OA

**Figure 2:**
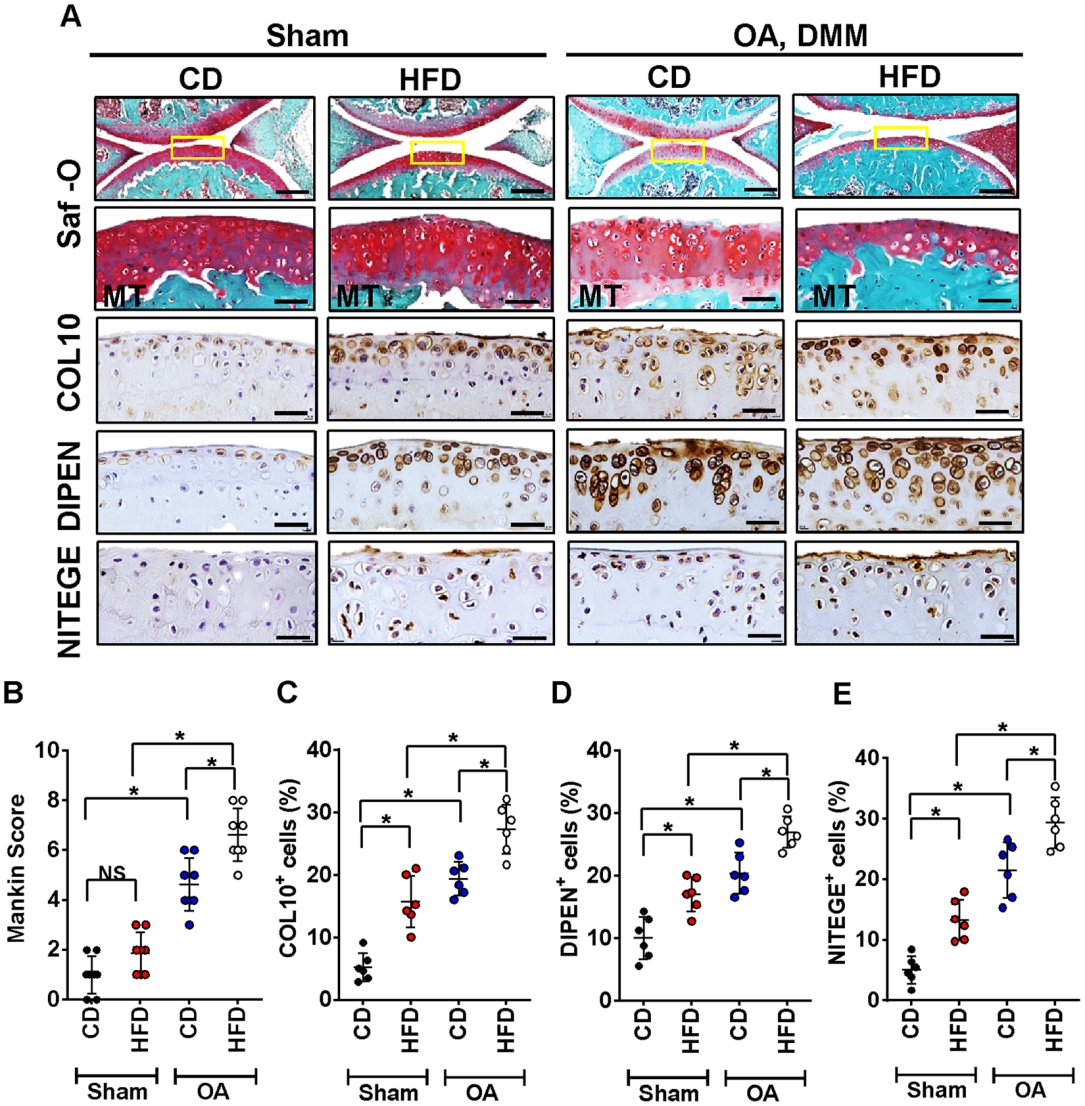
High-fat diet induces OA-like cartilage changes and accelerates surgically-induced OA. (**A**) Top panel: Representative Safranin O and fast green stained sagittal sections of sham or experimental OA knee regions in mice fed a CD or HFD. Scale bars, 100 µm. The inset boxes in upper re shown at higher resolution in lower panels. Scale bars, 100 µm. Bottom panel: Similar sections were stained with COL10, DIPEN, and NITEGE to determine if HFD resulted in OA-like cartilage molecular changes. Scale bars, 100 µm. (**B**) Severity of articular cartilage degradation was graded using Mankin scoring system. Graphs represent mean ± SD (n = 7). *p < 0.05. (C-E)The percentage of COL10 (**C**), DIPEN (**D**), and NITEGE (**E**) - positive cells per knee section were counted. Graphs represent mean ± SD (n = 6). *p < 0.05. Saf-O: Safranin O and fast green staining; MT: medial tibia.

Next we sought to determine whether HFD led to OA-like changes in articular cartilage. HFD administration showed non-significant histomorphometric appearances of cartilage as compared to CD-fed mice (Fig. 2). In surgically-induced OA mice, a progressive worsening of OA was observed in both HFD and CD-fed mice (Fig. 2A). However, OA was more severe in HFD-fed mice compared to CD-fed mice with post-surgery, as indicated by Mankin scoring (Fig. 2B). Immunohistochemical analysis of OA marker expression was used to determine if HFD resulted in OA-like cartilage changes. As shown in Figs 2A,C–E, in contrast to Mankin score, HFD-fed mice showed a significant increased expression of COL10-, DIPEN-, NITEGE-positive articular chondrocytes in knee joints compared to CD-fed mice at the same time points. Both surgically-induced OA groups displayed quantitatively more COL10, DIPEN and NITEGE positive cells (Fig. 2C–E). However, cartilage degradation was significantly greater in OA-HFD-fed mice compared to OA-CD-fed mice.

### Inflamed synovial joints contain M1 macrophages in high fat diet mice

**Figure 3:**
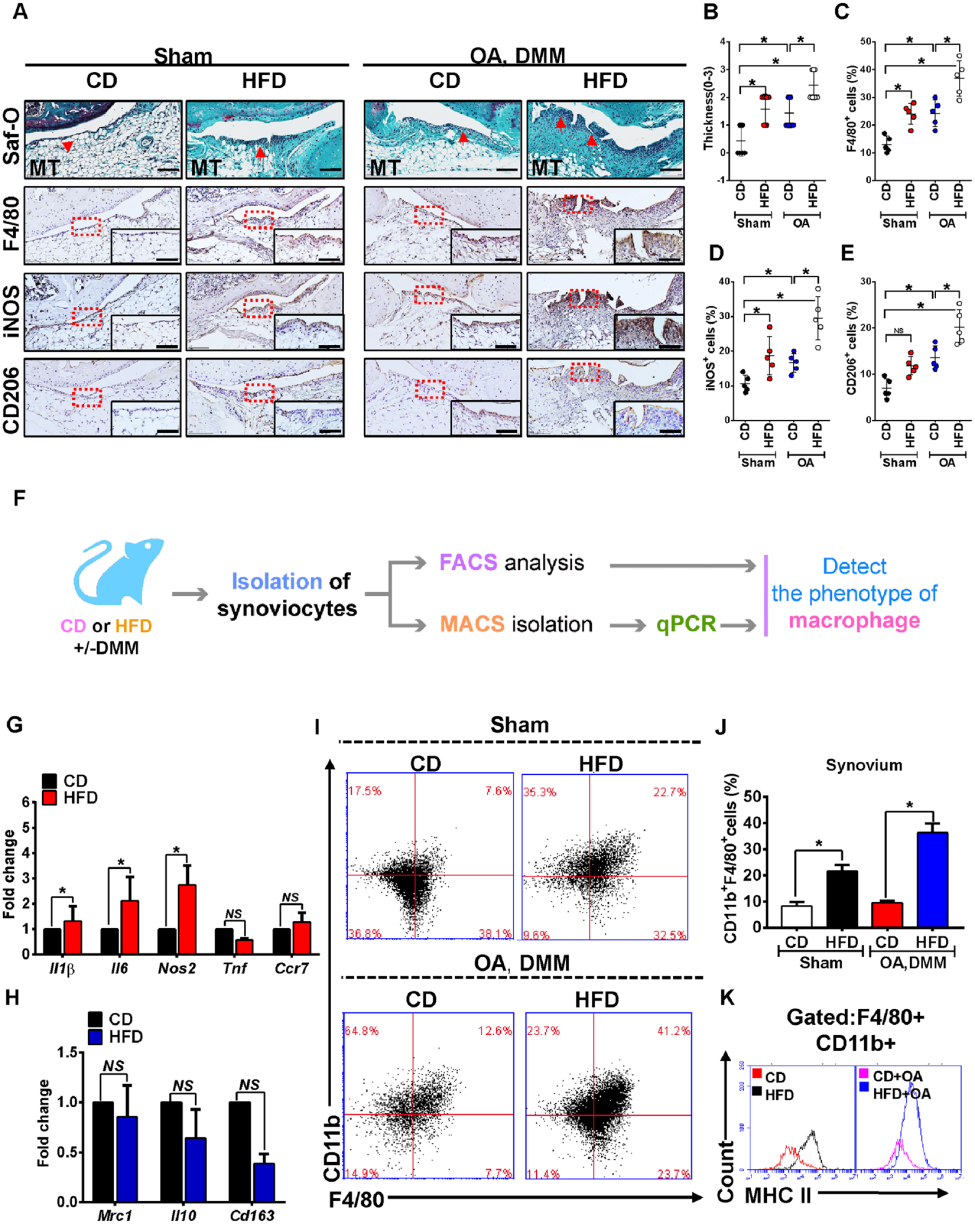
Inflamed synovium expresses a dominant M1 signature during the challenge of High-fat diet. (**A**)Top panel: Representative Safranin O and fast green stained sagittal sections of sham or experimental OA knee regions in mice fed a CD or HFD diet. Scale bars, 100 µm. Bottom panel: Similar sections were stained with F4/80, iNOS, and CD206 to determine the phenotype of synovial macrophage in activated synovium from CD- or HFD-fed mice. Scale bars, 100 µm. Insets are enlarged images of stained sections. (**B**) Synovial inflammation was assessed using synovitis scoring based on degree of cell thickness in the synovial lining layer and cell density of the synovial stroma. Graphs represent mean ± SD (n = 7). *p < 0.05. (**C**–**E**)The percentage of F4/80 (**C**), iNOS (**D**), and CD206 (**E**) - positive cells per knee section were counted. Graphs represent mean ± SD (n = 5). *p < 0.05. (**F**) Schematic diagram showing the experimental procedure, from the isolation of synoviocytes from animals to analyse the phenotype of synovial macrophage in the synovium from multiple biopsies of CD- and HFD-fed mice. qPCR analysis of pro-inflammatory M1-like (**G**) or anti-inflammatory M2-like (H) genes in MACS isolated F4/80+ synovial macrophage from CD- or HFD-fed mice. (**I**–**J**) FACS analysis of synovial macrophage from CD- or HFD-fed mice. (**K**)Same biopsies were further stained with MHC II to analyse the population of M1 activated macrophage. Saf-O: Safranin O and fast green staining; MT: medial tibia.

Histological assessment of synovium demonstrated increased cell density and lining-layer thickness in HFD-fed mice compared with the respected CD-fed mice (Fig. 3A). The occurrence of synovial fibrosis was also observed in HFD-fed mice. Thus, the synovitis score was significantly higher than that observed in control (Fig. 3B). The combination of OA surgery with high-fat diet induced worse synovial inflammation as determined by synovitis scoring (Fig. 3A,B). The quantity of F4/80^+^ macrophages was higher in inflamed synovium of mice treated with HFD, including those with surgically induced OA. These macrophages displayed significant changes towards a pro-inflammatory M1 phenotype (iNOS+), with no significant changes in the anti-inflammatory M2 macrophages (CD206+) (Fig. 3A–E). Furthermore, pro-inflammatory M1 macrophage markers interleukin (*Il*)*1β*, *Il6* and *Nos2* were significantly higher in MACS isolated F4/80^+^ synovial macrophages from HFD-fed mice compared with CD-fed mice. However, HFD did not significantly alter the expression of tumour necrosis factor (*Tnf*) and C-C chemokine receptor type 7 (*Ccr7*) (Fig. 3G). Conversely, expression of anti-inflammatory M2 macrophage markers *Mrc1* (CD206), *Il10* and *Cd163* was not different between obese and non-obese mice (Fig. 3H). In order to confirm the above observation, FACS analysis was performed (Fig. 3F,I–K). HFD significantly altered the percentage of CD11b^+^F4/80^+^ synovial macrophages from obese mice (Fig. 3I–J), and it was associated with higher surface expression of major histocompatibility complex call II (MHC II^high^) molecules (Fig. 3K), indicative of highly differentiated and activated macrophages.

### Clodronate liposome delivery attenuates cartilage damage and synovial inflammation induced by HFD in a post-traumatic OA model

**Figure 4:**
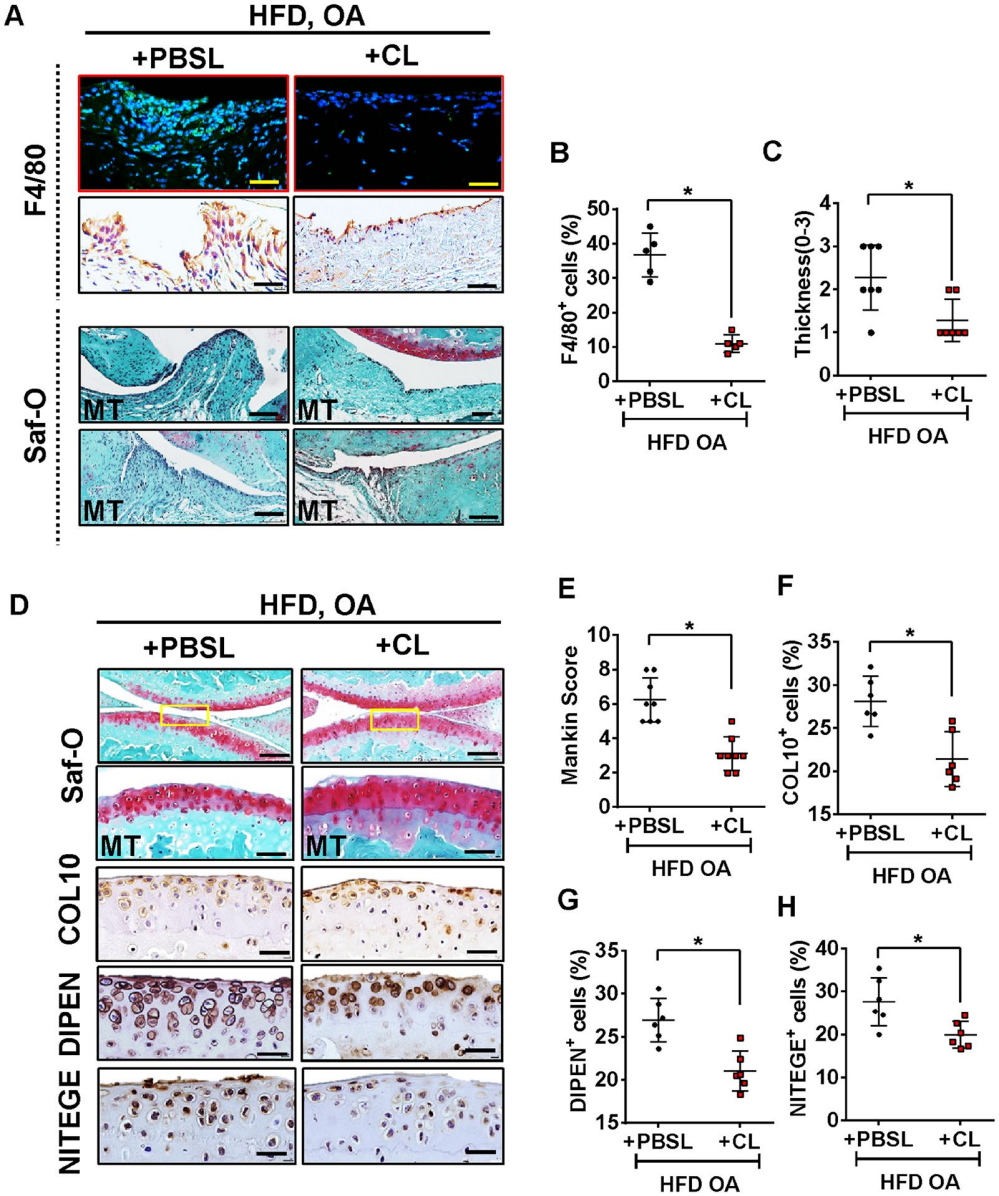
Clodronate liposome attenuates cartilage damage and synovial inflammation induced by High-fat diet in post-traumatic OA model. (**A**) Top panel: Knee sections were stained with F4/80 to determine the effect of Clodronate liposome on synovial macrophages. Scale bars, 100 µm. Bottom panel: Representative safranin-O image showed treatment effects from Clodronate liposome in the synovium of HFD-fed mice with surgically-OA. Scale bars, 100 µm. (**B**) The percentage of F4/80-positive cells per knee section were counted. Graphs represent mean ± SD (n = 5). *p < 0.05. (**C**) Synovial inflammation was assessed using synovitis scoring based on degree of cell thickness in the synovial lining layer and cell density of the synovial stroma. Graphs represent mean ± SD (n = 7). *p < 0.05. (**D**) Top panel: Representative Safranin O and fast green stained sagittal sections of experimental OA knee regions in mice fed HFD diet after Clodronate liposome treatment. Scale bars, 100 µm. The inset boxes in upper re shown at higher resolution in lower panels. Scale bars, 100 µm. Bottom panel: Similar sections were stained with COL10, DIPEN, and NITEGE to determine if Clodronate liposome attenuated cartilage damage. Scale bars, 100 µm. (**E**) Severity of articular cartilage degradation was graded using Mankin scoring system. Graphs represent mean ± SD (n = 8 ). *p < 0.05. (F-H) The percentage of COL10 (**F**), DIPEN (**G**), and NITEGE (**H**) - positive cells per knee section were counted. Graphs represent mean ± SD (n = 6). *p < 0.05. PBSL: PBS liposome; CL: Clodronate liposome. Saf-O: Safranin O and fast green staining; MT: medial tibia.

To determine the effect of synovial macrophages on the development of OA-like changes in HFD mice, we locally depleted macrophages by intra-articular injection of CL. We observed that CL administration resulted in a significant reduction in the number of F4/80^+^ synovial macrophage in HFD-OA mice compared to untreated group (Fig. 4A,B). We investigated if CL treated HFD regimes exhibited any effect on the degree of synovitis in surgery-induced OA. As shown in Fig. 4A, histological investigations revealed that affected synovium in the CL-treated HFD-fed mice showed significant decreases in membrane thickness and influx of synoviocytes. The synovitis scores reiterated these observations with a lower score in CL treated HFD-fed mice subjected to DMM surgery (Fig. 4C).

Given that CL treatment lowered synovial inflammation, we further determined whether the changes of synovium had protective effect on cartilage. The absence of synovial macrophage in the OA mice on HFD resulted in an increased content of proteoglycan compared with untreated OA mice fed with HFD (Fig. 4D). Also noteworthy was the observation that OA severity, graded using Mankin score, was significantly lower in the CL-treated HFD-OA mice relative to the PBSL (vehicle) treated group (Fig. 4D,E). Moreover, CL-treated HFD-OA mice showed reduced expression of COL10, DIPEN and NITEGE (Fig. 4D,F–H). As shown in Figure Supplementary Fig. 1, a slight increase in the Mankin scores was observed in the cartilage of CL-treated mice on CD compared with vehicle-treated CD-fed mice. Additionally, the CL-treated mice exhibited increased expression of COL10, DIPEN and NITEGE (Supplementary Fig. 1A–E).

With respect to metabolic factors, local intra-articular injection of CL did not affect body weight of HFD-fed mice (Supplemental Fig. 2A). However, with the exception of serum resistin levels which were decreased in HFD-fed mice treated with CL vs. HFD-fed mice without CL (Supplemental Fig. 2B), the levels of leptin and adiponectin levels were not altered by CL treatment (Supplemental Fig. 2C,D). Serum insulin levels in CL treated HFD-fed mice were likewise not reduced (Supplemental Fig. 2E). As shown in Supplemental Fig. 2F,G, no effects on lipid profiles including triglyceride and total cholesterol were observed in CL-treated HFD mice.

### Resolvin D1 treatment enhance the resolution of synovial inflammation

**Figure 5:**
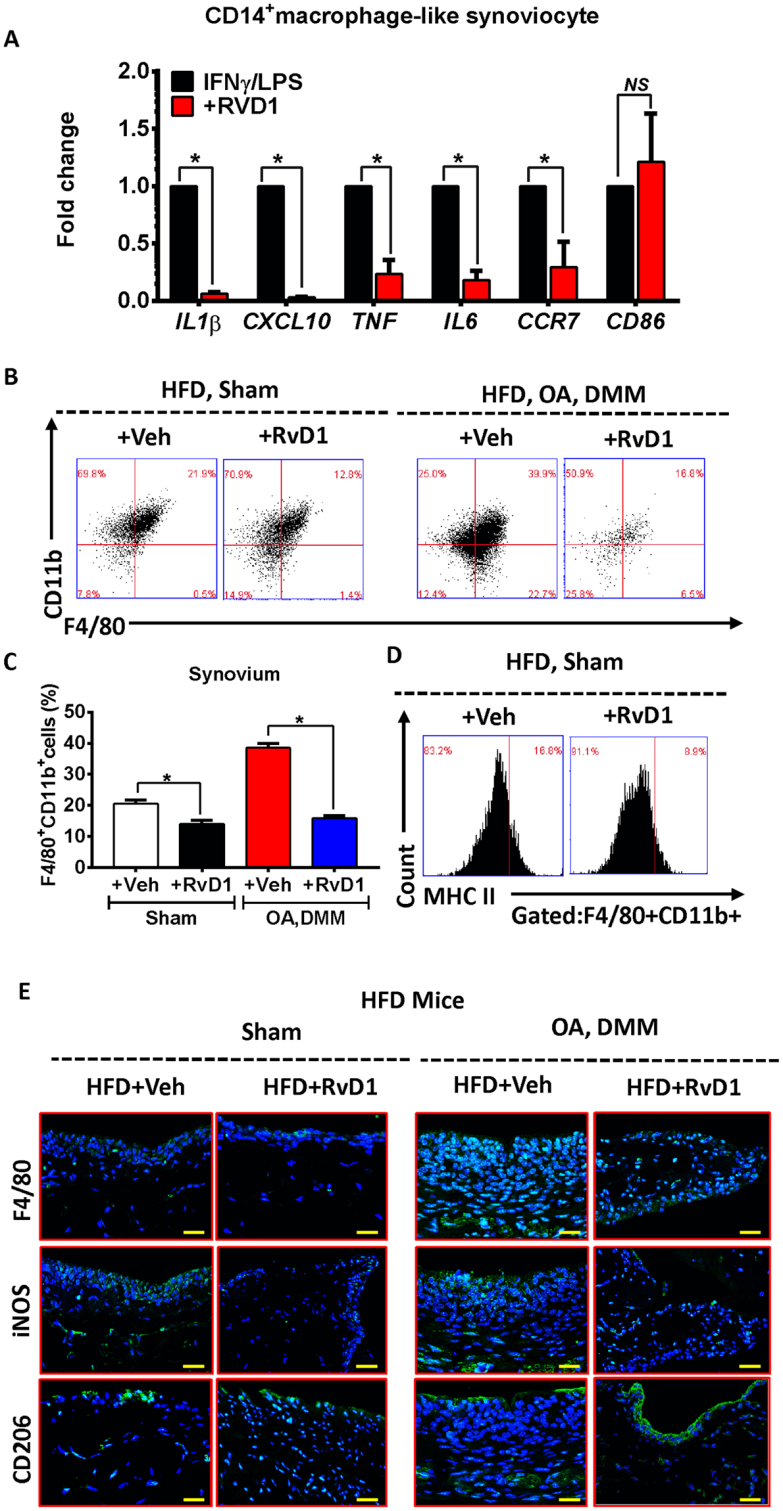
Resolvin D1 treatment improves the resolution of synovial inflammation. (**A**) MACS isolated human CD14+ synovial macrophage were stimulated to M1 phenotype and then treated with RvD1 for 24 h, qPCR analysis of pro-inflammatory M1-like genes were performed *in vitro*. Graphs represent mean ± SD (n = 4). *p < 0.05. (**B**,**C**) FACS analysis of synovial macrophage from CD- or HFD-fed mice with or without OA after RvD1 treatment. (**D**) Same biopsies were further stained with MHC II to analyse the population of M1 activated macrophage. (**E**) Knee sections were stained with F4/80, iNOS, and CD206 to determine the phenotype of synovial macrophage in activated synovium from HFD-fed mice with or without OA after RvD1 treatment. Scale bars, 100 µm. RvD1: resolvin D1; Veh: placebo (1% ethanol in saline).

After identification of the distinct contribution of M1 polarised synovial macrophages in the development of HFD induced OA, we next sought to determine if pharmacological intervention using RvD1 could alleviate the pro-inflammatory phenotype of macrophages in OA. We first investigated whether RvD1 might modulate human M1 macrophages *in vitro*. Treatment of M1-polarised CD14^+^ macrophages with RvD1 strongly suppressed the expression of M1 macrophage markers *Il1β*, *Cxcl10*, *Tnf*, *Il6* and *Ccr7*, with no significant decrease in expression of co-stimulation marker *Cd86* (Fig. 5A). These results indicate RvD1 treatment can decrease pro-inflammatory markers. To determine whether the anti-inflammatory properties of RvD1 in the synovium were related to changes in the population of synovial macrophages *in vivo*, these cells were characterised by flow cytometry. As shown in Fig. 5B,C, the administration of RvD1 to HFD mice markedly reduced the number of macrophages (F4/80^+^CD11b^+^) in synovium of sham and surgically-induced OA models. Notably, the population of activated macrophages (F4/80^+^CD11b^+^MHC II^high^) was decreased in RvD1-treated HFD mice (Fig. 5D).

To further determine whether the phenotype of synovial macrophages was modulated by RvD1 treatment, we isolated synovium from sham and surgically induced OA mice on HFD treated with or without RvD1, and determined F4/80, iNOS and CD206 tissue expression. As shown in Fig. 5E, the treatment of RvD1 reduced the number of F4/80^+^ macrophages in HFD fed mice even in those with surgically induced OA. The number of M1 macrophages (iNOS^+^) was significantly suppressed after treatment. In contrast to M1, the number of M2 macrophages (CD206^+^) was significantly higher in RvD1 treated HFD mice compared with vehicle-treated groups (Fig. 5E), suggesting that RvD1 treatment enhanced the resolution of synovial inflammation.

### Resolvin D1 treatment reduces the severity of HFD induced OA

**Figure 6:**
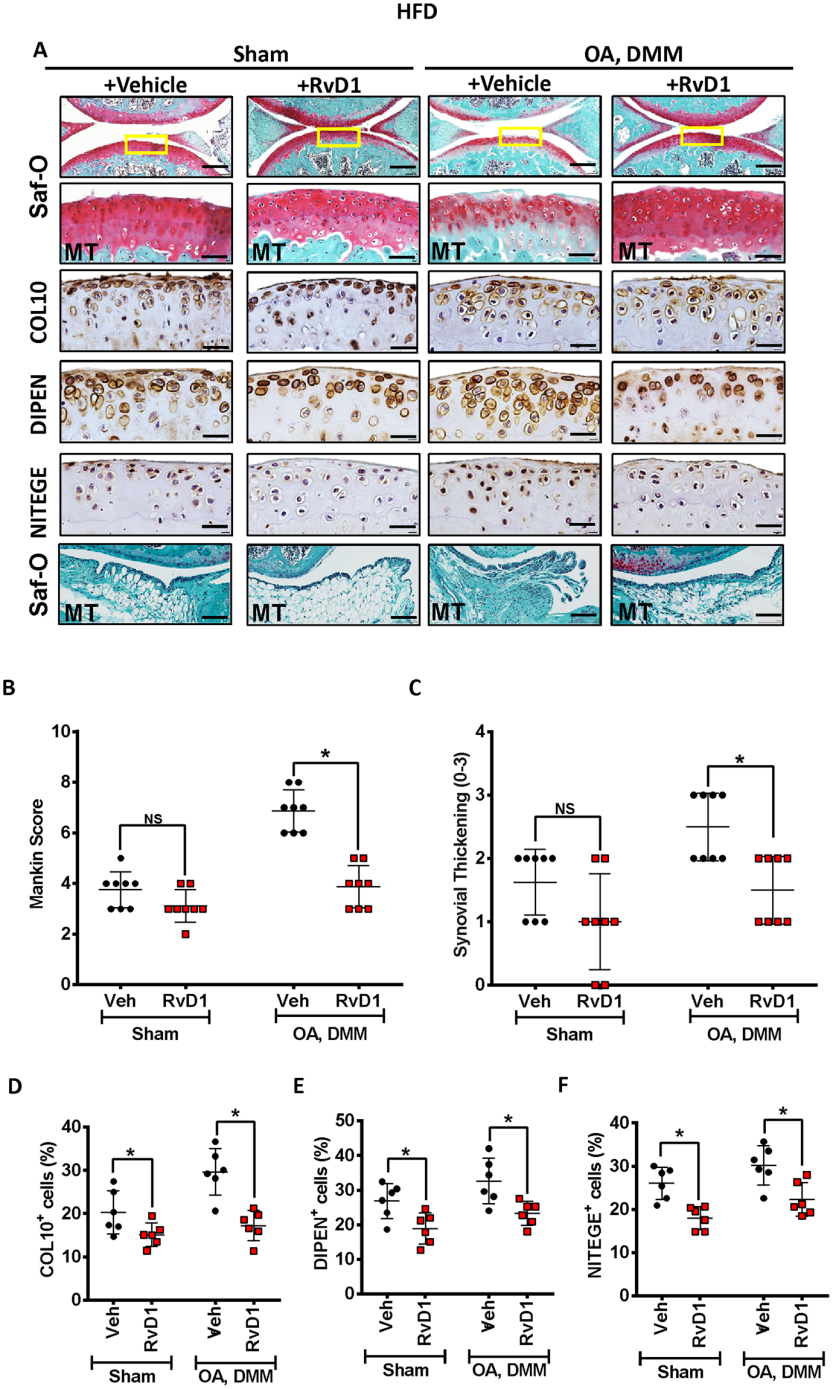
Resolvin D1 treatment reduces the severity of HFD in surgically induced OA. (**A**) Top panel: Representative Safranin O and fast green stained sagittal sections of sham or experimental OA knee regions in mice fed a HFD diet with or without RvD1 treatment. Scale bars, 100 µm. The inset boxes in upper re shown at higher resolution in lower panels. Scale bars, 100 µm. Bottom panel: Similar sections were stained with COL10, DIPEN, and NITEGE to determine the effect of RvD1 on cartilage. Scale bars, 100 µm. Representative safranin-O image show treatment effects of RvD1 in the synovium of HFD-fed mice with surgically-OA. Scale bars, 10 µm. (**B**) Severity of articular cartilage degradation was graded using Mankin scoring system. Graphs represent mean ± SD (n = 8). *p < 0.05. (**C**) Synovial inflammation was assessed using synovitis scoring based on degree of cell thickness in the synovial lining layer and cell density of the synovial stroma. Graphs represent mean ± SD (n = 8). *p < 0.05. (**D**–**F**)The percentage of COL10 (**D**), DIPEN (**E**), and NITEGE (**F**) - positive cells per knee section were counted. Graphs represent mean ± SD (n = 6). *p < 0.05. RvD1: resolvin D1; Veh: placebo (1% ethanol in saline). Saf-O: Safranin O and fast green staining; MT: medial tibia.

In view of RvD1’s protective actions in resolution of synovial inflammation, next we tested its effect on OA cartilage severity under obese conditions. In response to RvD1 treatment, HFD-OA mice showed less evidence of cartilage degradation compared to untreated mice, such as proteoglycan loss and cartilage surface irregularities compared with the untreated OA animals (Fig. 6A). The Mankin scores reiterated these observations with a lower score in both RvD1 treated HFD-OA mice (Fig. 6B). Histologic analysis of RvD1 treated HFD-OA mouse model showed significantly decreased synovial thickening compared with untreated group (Fig. 6A,C). Immunohistochemical staining of cartilage specimens showed lower expression of COL10, DIPEN and NITEGE in both RvD1-treated animals fed HFD (Fig. 6A,D–F).

We further performed biochemical analyses to detect the metabolic parameters in RvD1 treated animals. As shown in Supplementary Fig. 3A, no effect on body weight were observed in RvD1 treated HFD fed mice. Except for serum leptin, resistin and adiponectin levels were improved in HFD-fed mice treated with RvD1 (Supplementary Fig. 3B–D). Intra-articular injection of RvD1 also led to an improvement in serum insulin level, although this was not significantly different compared to HFD-fed mice (Supplementary Fig. 3E). Furthermore, serum triglyceride and total cholesterol levels were similar between RvD1 treated and untreated HFD-fed mice (Supplementary Fig. 3F–G).

## Discussion

Considerable epidemiological data links obesity with an increased likelihood of OA development. Although several studies suggest that diets high in fat content increase the risk of OA development and inflammation, the mechanisms have not been elucidated. In this study, using relevant murine models of obesity and OA, we show that (1) HFD increases the rate of progression of OA in a post-traumatic OA mouse model, (2) the metabolic effects of HFD are driven in part by synovial infiltration of pro-inflammatory macrophages, (3) that depletion of macrophages using clodronate-liposomes may partially rescue the obesity-associated post-traumatic OA, and (4) resolution of inflammation by RvD1 intervention can attenuate the effects of HFD on OA.

In our experiments, C57BL/6J mice on a HFD regimen (45% kcal fat) alone failed to show any significant Mankin score changes compared to aged-matched mice on the control diet of 10 kcal% fat. These findings are in corroboration with a recent study which demonstrates that diet-induced metabolic dysfunction per se does not lead to aggravated articular cartilage degradation in mice^9^. In the presence of an additional trigger such as post-traumatic OA, we observed significant changes in the cartilage damage and increase in the Mankin scores in HFD group. Similar to this observation, Wu *et al*. showed that high-fat diet accelerated the progression to OA after destabilization of the medial meniscus^10^. Similarly, Datta *et al*. and Louer *et al*. showed that in addition to increased body weight and changes to metabolism, HFD also contributed to the accelerated progression of OA in mice^11,12^. Taken together, these results suggest that a high-fat diet plays a contributing role to acceleration of OA.

Previous reports have shown the infiltrating immune cells in OA synovial tissues include macrophages, T cells, and to a minor extent, mast cells and B cells^13–15^. In this study, we observed that HFD aggravated OA synovitis, by inducing severe tissue architecture disorganisation of the synovium, along with infiltration of macrophages both in the presence and absence of post-traumatic OA. It is interesting to note that although HFD alone did not significantly induce cartilage damage, it causes changes to synovitis. These data are in line with previously published result and possibly suggest that the aggravation in synovial inflammation induced by HFD is not a secondary event resulting from a pathological change in the cartilage^16^. In obesity, the percentage of macrophages in adipose tissue can be as high as 50% of all adipose tissue cells while in lean individuals macrophages constitute around 5% of the cells^17^. Of importance, as well as increasing in number, these macrophages display a unique inflammatory phenotype during obesity^18^. Our findings indicate that synovium and fat tissue surrounding the knee joint mitigate the same pattern of macrophage polarisation leading to a pro-inflammatory status in obese conditions. However, in this study we were unable to define the origin of these macrophages. Although it is believed that accumulated adipose tissue macrophages is caused by monocyte migration under obesity-induced inflammatory condition, the tissue-resident macrophages are capable self-renewal and proliferate locally^19,20^. Moreover, a recent study showed that both local proliferation and migration of macrophages is responsible for adipose tissue macrophage infiltration during obesity development^21^. Therefore, both local proliferation and migration of macrophages might contribute to synovial macrophage accumulation during development of obesity-associated OA.

Next, we determined whether macrophages play a role in promoting the development of synovitis and cartilage degradation in obesity. Several previous studies have used clodronate liposomes to investigate the macrophage-mediated effects in different disease conditions. Studies have shown that clodronate was toxic when injected systemically and resulted in mortality in recipient mice, mainly due to cardiac complications^22^. Therefore, in our study clodronate liposomes were administered locally via intra-articular injections. Firstly, immunohistochemical staining against F4/80, further confirmed that significantly fewer macrophages were present in the HFD + DMM-operated joints treated with clodronate relative to liposome-only treated joints at the end of the experiment. This approach has marginally lowered the Mankin scores in HFD + OA treated compared to liposome-only treated controls, suggesting that macrophage-mediated tissue inflammation is a key component of association between obesity and OA. These findings are in contrast with Chia-Lung Wu *et al*., demonstrating that the conditional macrophage depletion increases inflammation and do not inhibit the development of OA in obese macrophage Fas-induced apoptosis-transgenic mice^23^. The differences between our findings and those of Chia-Lung Wu *et al*. may be due to several factors. Firstly, we used multiple injections and local depletion, while Chia-Lung Wu *et al*. used a short-term depletion approach which can lead to re-accumulation of inflammatory cells. In addition, the differences in the cartilage healing may be due to the extent of macrophage depletion in these tissues. Although cell type depletion models used in the study of Chia-Lung Wu *et al*. are a powerful tool in the analysis of the roles of macrophages, dendritic cells, neutrophils, and other immune cells, the consequences of systemic depletion can be significant and may have a confounding effect as they are immunocompromised due to lack of antigen presenting cell. For instance, depletion of professional antigen presenting cells like dendritic cells using CD11c-DTR mice results in neutrophilia which can have important roles in aggravation of inflammation and tissue destruction^24^. Also, it is known that chronic depletion of antigen presenting cells will change the microbiome that can influence the nutrient uptake such as short chain fatty acids etc. to exuberate immunopathology^25^.

Interestingly, in our study, we observed that selective depletion of synovial macrophages induced cartilage damage, as indicated by increased expression of COL10, DIPEN and NITEGE in cartilage of CD mice, such observation was not detected in animals following liposome-only treatment. However, under the challenge of HFD or HFD-OA, depletion of macrophages successfully decreased the severity of OA. These results indicate that in the normal joint microenvironment homeostatic macrophages may have important phagocytic and reparative functions and depletion of these macrophages can have off-target effects; however in conditions such as those in obesity and OA, prolonged macrophage responses are thought to exacerbate the injury by preventing the resolution of inflammation.

Next, we tested if modulating homeostasis of immune cells has beneficial effects in obesity-induced OA. RvD1 is mainly expressed on neutrophils and macrophages which is a derivate of DHA through a lipoxygenase (LOX) or aspirin-triggered cyclooxygenase-2 (COX-2) pathway with potent anti-inflammatory and pro-resolving properties^26,27^. Previous studies have shown that RvD1 can regulate the macrophage polarisation, by switching gene regulation profiles from pro-inflammatory M1 type macrophages into pro-resolution M2 macrophages via two G-protein-coupled receptors, ALX/FPR2 and GPR32^28,29^. In our study, RvD1 treatment modify macrophages from an MHC II^high^ to a MHC II^low^ phenotype and decreased the population of CD11b^+^F4/80^+^ macrophages in synovium which results in reduced synovium thickening. In addition to synovium changes, we also observed that intra-articular treatment of RvD1 on HFD mice showed less evidence of cartilage degradation compared to untreated mice. This was consistent with the previous study that demonstrated RvD1 stifled IL-1β induced catabolic metabolism in OA chondrocytes *in vitro* by suppressing NF- Κb/p65, p38/MAPK and JNK1/2^30^. Many different signalling molecules exist that are regulated by RvD1, and these candidate markers represent an interesting area for future mechanistic studies.

In conclusion, obesity induces an accumulation of pro-inflammatory macrophages in the synovium and fat pad tissues. The resulting resident macrophage population establishes a pro-inflammatory environment that enhances OA development and pathology. Furthermore, RvD1 treatment reduces pro-inflammatory gene expression, increases anti-inflammatory gene expression, and ultimately induces M2 macrophage polarisation to mitigate the effects of obesity-associated OA development.

## Methods

### Study Approval

Experiments were performed in accordance with protocols approved by the Institutional Animal Care and Use Committees and Institutional Biosafety Committees of both Queensland University of Technology (University Animal Ethics Committee UAEC # 1600000254) and Central–South University (2013–05). For experiments involving human tissue samples, ethical approval was granted by the Queensland University of Technology and The Prince Charles Hospital Ethics Committees (Approval number #1400001024). Informed consent was obtained from all human subjects.

### Animal experimental protocols

Six-week-old male C57BL/6 mice were purchased from the Animal Centre of Central–South University (Changsha Shi, Hunan Sheng, China), and the Animal Resources Centre (Perth, WA, Australia). Mice were fed with either standard rodent chow [control diet (CD)] containing 10 kcal% fat, 20 kcal% protein and 70% carbohydrate or western-style diet (HFD) containing 45 kcal% fat, 20 kcal % protein and 35 kcal% carbohydrate (Specialty Feeds, Glen Forrest, WA, Australia or Research Diets, Biopike, Shanghai, China). Our lab, as well as others, has implemented the aforementioned high-energy diet as a surrogate for the HFD. After 8 weeks of feeding, a group of mice randomly underwent DMM surgery to induce OA. To induce OA in the mice, the left knee was opened using a medial parapatellar approach, and the medial meniscal ligament was dissected to achieve destabilisation of the medial meniscus of the left knee joint, as previously described^31^. All the mice with or without DMM were maintained on the same diets until experimental endpoints at 22-week of age. Mice were group housed (5 animals/cage) in ventilated double-decker cages in a temperature-controlled room on 12-h light/dark cycles with *ad libitum* access to food and water and routine veterinary assessment. Body weight was measured each week and blood samples were taken to assess physiological measurement and metabolic parameters.

### Macrophage depletion by injection of Clodronate liposomes

To investigate the effects of macrophages on synovial inflammation and cartilage degradation, one week prior to surgery, mice in experimental OA HFD group were administered 0.05 mg of Clodronate liposomes or PBSL controls (Encapsula Nano Sciences LLC, Brentwood, TN, America) by intra-articular injection. All experimental mice were injected one week prior surgery and on week 1 and week 6 after surgery and euthanized at 8 weeks post-surgery. Intra-articular injection was performed through the patellar tendon with the length of the needle adjusted to 2.4 mm with volume of 10 μL.

### Treatment with pro-resolving factor Resolvin D1

One week prior to experimental OA-induction, obese mice were treated with 20 ng/µl Resolvin D1 (Cayman Chemical, Redfern, NSW, Australia) or placebo (1% ethanol in PBS) by intra-articular injection in 10 μL volume. All experimental mice were injected one week prior surgery and on week 1 and week 6 after surgery in the OA model and euthanized at 8 weeks post-surgery and the knee joint tissues were harvested.

### Biochemical analyses

Mice were sacrificed, blood was withdrawn through cardiac puncture and the serum was collected for biochemical analysis. Serum leptin, resistin, adiponectin, and insulin were measured using enzyme-linked immunosorbent assay (ELISA) kit following the manufacturer’s instructions (Abcam, Changsha, Hunan, China). Serum total cholesterol (TC), high-density lipoprotein cholesterol (HDL-c) and low-density lipoprotein cholesterol (LDL-c) were using a Cholesterol Assay Kit (Abcam). The concentration of serum triglyceride was determined using a Triglyceride Quantification Kit (BioVision, Dakewe Biotech, Beijing, China).

### Histological assessment of osteoarthritis development in mice

After euthanasia, the knee joints were harvested and fixed overnight in 4% paraformaldehyde in 1X PBS, decalcified in 10% ethylenediaminetetraacetic acid, embedded in paraffin and cut with a rotary microtome to generate 5 micrometer-thick sagittal sections. Sections were stained with fast green and Saf-O and then evaluated for cartilage damage and synovial inflammation by two independent assessors under blinded conditions. Cartilage OA-like change was scored using a Mankin score and synovial thickening was assessed using a 0–3 scoring system as previously described^32,33^ (0 = no synovial thickening; 1 = lining of two cell layers; 2 = several extra cell layers; 3 = clear inflammation with cell infiltrate). Immunohistochemistry was performed according to our previously published study with modifications to determine the synovial macrophage phenotypes: anti-F4/80 (Abcam; Cat No: ab6640, Melbourne, VIC, Australia; dilution 1:50), anti-iNOS (Abcam; Cat No: ab15323 dilution 1:100) and anti-CD206 antibodies (Abcam; Cat No: ab6469, dilution 1: 50). Anti-Aggrecan NITEGE epitope (kind gift from Professor Amanda Fosang, Murdoch Children Research Institute, Melbourne, VIC, Australia; dilution 1:900), anti-collagen DIPEN neoepitope (kind gift from Professor Amanda Fosang, Murdoch Children Research Institute, Melbourne, VIC, Australia; dilution 1:1200), and anti-type X collagen (Abcam; Cat No: ab58632, dilution 1:800) antibodies were used to determine the expression in sections were detected using immunohistochemistry. The sections were incubated with corresponding fluorescent secondary antibodies. Immunofluorescence was examined with a Leica SP5 confocal microscope. To conduct semi-quantitative data analysis, the positive cells within cartilage in each field (40× objective lens) of observation were counted and normalised to the cell number per 100 total cells in each group using ImageJ (National Institute of Health, Bethesda, BA, USA).

### Isolation of synoviocytes and macrophage quantification using FACS analysis

Immediately after euthanasia, the skin of the hind limbs was removed and synovial tissues around the knee joints were pooled from 4 animals per group as previously described^34–36^. The harvested tissues were minced using sterile scalpel and digested in a 1 mg/ml collagenase type I (Sigma) for one hour at 37 °C and rinsed through a 70-µm filter (BD Biosciences, North Ryde, NSW, Australia)^34–36^. The cells were suspended in phosphate-buffered saline (PBS) containing 20 µg/ml of antibodies. Fluorescein isothiocyanate (FITC)-, phycoerythrin (PE)- or allophycocyanin (APC)-conjugated antibodies against mouse F4/80, CD11b and MHCII was obtained from BD Biosciences. For isotype controls, FITC-, PE-, APC- conjugated nonspecific rat immunoglobulin G was substituted for the primary antibody. After incubating with antibody cocktails for 30 minutes at 4 °C, the cells were washed with PBS and resuspended in PBS for the analysis. Data were acquired on a BD Accuri C6 cytometer (BD Biosciences, North Ryde, NSW, Australia) and analysis of the data was performed using BD Accuri C6 1.0 software (BD Biosciences, North Ryde, NSW, Australia).

### Isolation of macrophages from mouse synovium

Synoviocytes were isolated from four mice per group by digestion with type 1 collagenase as described above. Synovial macrophages were purified using anti-F4/80 magnetic beads. Synoviocytes were suspended in 90 µl PBS per 10^7^ total cells containing anti-F4/80 UltraPure microbeads (Miltenyi Biotec, Macquarie Park, NSW, Australia) and then incubated for 30 min at 4 °C in the dark according to the manufacturer’s instruction. The F4/80 magnetically labelled cells were purified by magnetic activated cell sorting (MACS, MASC Separation columns MS; Miltenyi Biotec, Macquarie Park, NSW, Australia). The collected F4/80-positive cells were centrifuged at 300 *g* for 10 min. The supernatants were removed and cell pellets were then used directly for RNA isolation. Total RNA from harvested macrophage-like synoviocytes were extracted using RNeasy Mini kit (Qiagen, Doncaster, VIC, Australia) according to the manufacturer’s instructions.

### Human synovium tissue collection and preparation

Human synovium being discarded as medical waste were obtained from seven consenting patients (average age = 68.3 ± 4) with OA (Kellgren-Lawrence grade 4) immediately undergoing total knee replacement at The Prince Charles and Holy Spirit Northside Private Hospital, Brisbane, QLD. Explants were transferred to the lab in sterile cell culture media consisting of Dulbecco’s Modified Eagle’s Medium (DMEM) supplemented with 1% penicillin-streptomycin. Synovium biopsies were cleaned of extraneous fat prior to isolation of macrophages.

### Effect of resolvin D1 (RvD1) on human synovial macrophages

OA synovium were isolated and digested as previously described^34–36^. To prepare a population of synovial macrophages, the cells were suspended in 80 µl PBS supplemented with 0.5% bovine serum albumin (Sigma-Aldrich) containing anti-human CD14 microbeads (Miltenyi Biotech) per 107 total cells, and then incubated for 20 min at 4 °C in the dark according to the manufacturer’s instruction. The cells were sorted into a CD14-positive cell fraction and a CD14-negative cell fraction. The magnetic separation procedure was repeated by using a new column to increase the purity of CD14-positive cells. Freshly isolated human CD14-positive macrophages were suspended in low-glucose DMEM supplemented with 1% penicillin-streptomycin and 10% heat-inactivated foetal bovine serum at 37 °C for 24 hours in a 24-well cell culture plate at a density of 0.5 × 106 per well. Non-adherent cells were removed by washing twice with PBS and the remaining adherent cells were used for the experiments. To assess M1 macrophage phenotype, resting cells were stimulated with either (1) 100 ng/ml LPS from E.coli (Sigma-Aldrich, Castle Hill, NSW, Australia) plus 20 ng/ml recombinant human interferon (IFN)γ (R&D System, Noble Park, VIC, Australia) for M1 differentiation, or (2) no stimulation (control) at 37 °C for 24 hours, and then incubated for an additional 24 hour with resolvin D1 (1 nM) (Cayman Chemical) with modifications as previously described^37,38^. At the end of the incubation period, medium was removed and macrophages were washed twice and then collected in TRIzol (Invitrogen, Mt Waverley, VIC, Australia) and stored at −80 °C until RNA extraction was performed.

### RNA isolation and reverse transcription

Isolation of total RNA from CD14-positive macrophages and F4/80-positive macrophages was performed using TRIzol and RNeasy Mini kit, respectively. RNA quantity and quality was assessed in a NanoDrop-100 spectrophotometer (Thermo Scientific, Scoresby, VIC, Australia). cDNA synthesis from 260 to 400 ng of total RNA was performed using SensiFAST cDNA Synthesis Kit according to manufacturer’s protocol.

### Gene expression profiling by real-time PCR

For quantification of gene expression by real-time PCR, SYBR Green detection chemistry was used on the QuantStudio Real-Time PCR system (Applied Biosystems, Thermo Scientific, Scoresby, VIC, Australia). Quantitative measurements of all primers used in this study were determined using (2^−ΔΔCt^) method, and β-actin/GAPDH expression were used as the internal control, as described previously by our group^39–42^.

### Statistical analysis

Statistical difference of the results was tested using unpaired *t*-test (for two-group comparisons) or ANOVA method (for multi-group comparisons) followed by a *post hoc* test. All analyses were performed using GraphPad Prism 7 (San Diego, CA, USA) and *p*-values < 0.05 were considered to be significant. All data are presented as mean ± SD^6,7^.

## Supplementary information

Supplementary Information
