# Supplementary material for: Pro-resolving lipid mediator ameliorates obesity induced osteoarthritis by regulating synovial macrophage polarisation
Source: Sci Rep. 2019 Jan 23;9:426. doi: 10.1038/s41598-018-36909-9 (PMC6344566; doi:10.1038/s41598-018-36909-9)
Supplement: Supplementary file 1 — Supplementary Information [file 41598_2018_36909_MOESM1_ESM.pdf]

Pro-resolving lipid mediator ameliorates obesity induced osteoarthritis by regulating synovial macrophage polarisation

Antonia RuJia Sun [1], Xiaoxin Wu [3], Bohao Liu [3], Yang Chen [3], Charles W. Armitage[,4], Avinash Kollipara[4,5], Ross Crawford [1,2] , Kenneth W. Beagley[4], Xinzhan Mao [3*], Yin Xiao [1,6] and Indira Prasadam [1,6*]

[1]Institute of Health and Biomedical Innovation, Faculty of Science and Engineering, Queensland University of Technology, Brisbane, Australia, 4059
[2]The Prince Charles Hospital, Orthopedic Department, Brisbane, Australia
[3]Department of Orthopaedic Surgery, Second Xiangya Hospital, Central South University, Changsha, China
[4]Institute of Health and Biomedical Innovation, Faculty of Health, School of Biomedical Sciences, Queensland University of Technology, Brisbane, Australia
[5]Department of Pediatrics, School of Medicine, The University of North Carolina at Chapel Hill, Chapel Hill, USA.
[6]Australia–China Centre for Tissue Engineering and Regenerative Medicine, Queensland University of Technology, Brisbane, Queensland, Australia

***Corresponding author**
Indira Prasadam, Ph.D.
Email: i.prasadam@qut.edu.au

Prof Xinzhan Mao, MBBS, FRACS, DPhil.
E-mail: maoxinzhan72@126.com

Supplementary Figure 1

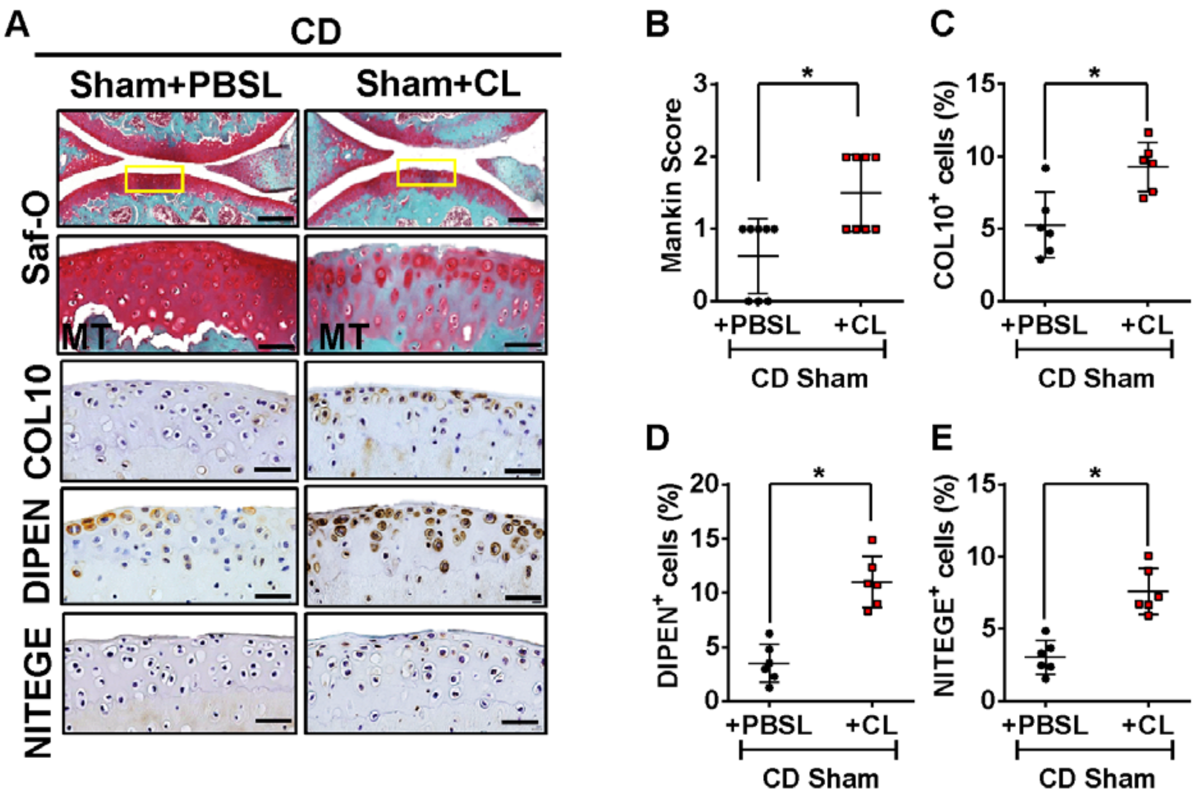

<u>Supplementary Figure 1:</u> Clodronate liposome causes cartilage damage in CD-fed mice. (A) Top panel: Representative Safranin O and fast green stained sagittal sections of knee regions in mice fed CD after Clodronate liposome treatment. Scale bars, 100μm. The inset boxes in upper re shown at higher resolution in lower panels. Scale bars, 100μm. Bottom panel: Similar sections were stained with COL10, DIPEN, and NITEGE to determine if Clodronate liposome attenuated cartilage damage. Scale bars, 100μm. (B) Severity of articular cartilage degradation was graded using Mankin scoring system. Graphs represent mean ± SD (n=8). *=$p < 0.05$. (C-E)The percentage of COL10 (C), DIPEN (D), and NITEGE (E)- positive cells per knee section were counted. Graphs represent mean ± SD (n=6). *=$p <$ 0.05. PBSL: PBS liposome; CL: Clodronate liposome. Saf-O: Safranin O and fast green staining; MT: medial tibia.

Supplementary Figure 2

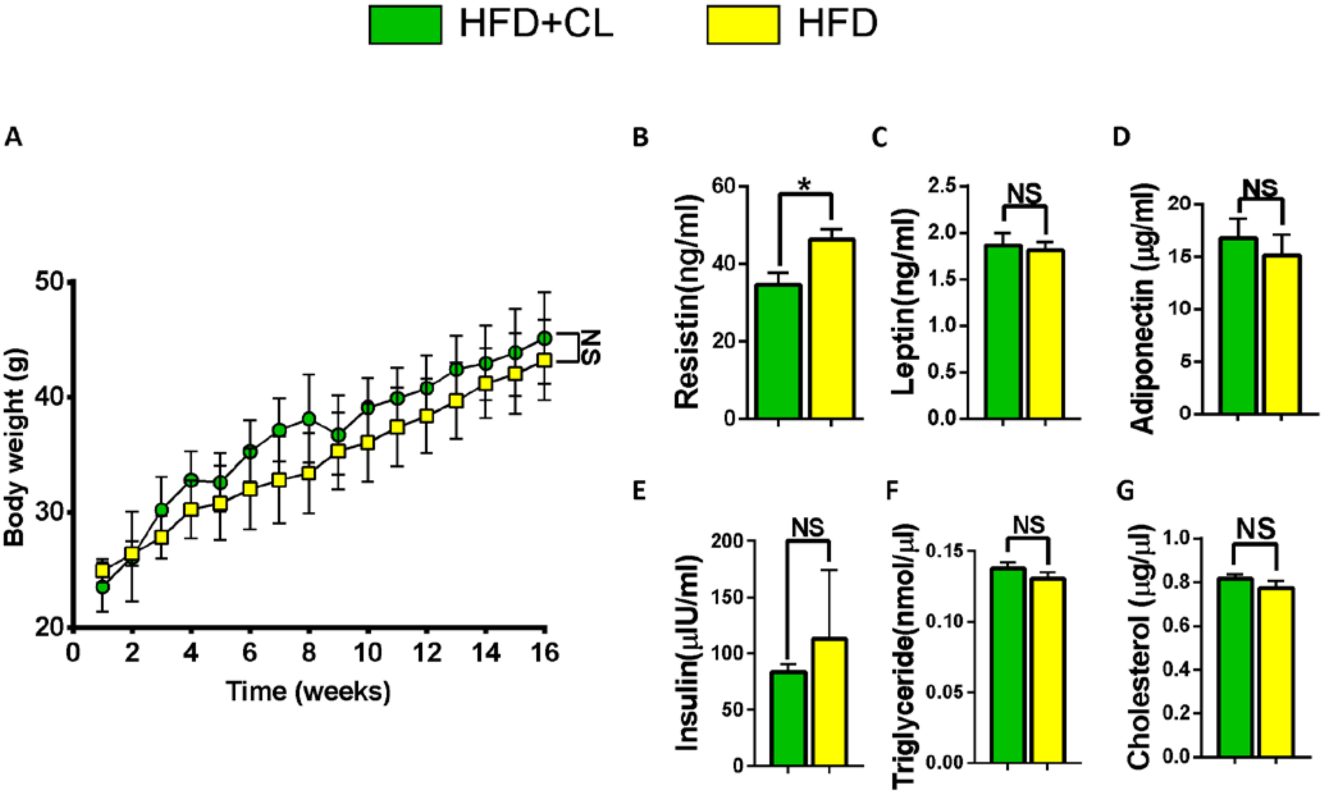

Supplementary Figure 2 : Local treatment of Clodronate liposome does not affect bodyweight but altered metabolic parameters. (A) Body weight of HFD+CL or HFD mice were monitored over 16 weeks. (B-J) Effect of CL on metabolic parameters. Measurement of serum resistin (B), leptin (C), adiponectin (D), insulin (E), triglyceride (G) and total cholesterol (H). Graphs represent mean ± SD (n=5). *=$p < 0.05$. HFD, High-fat diet-fed mice; CL, Clodronate liposome.

Supplementary Figure 3

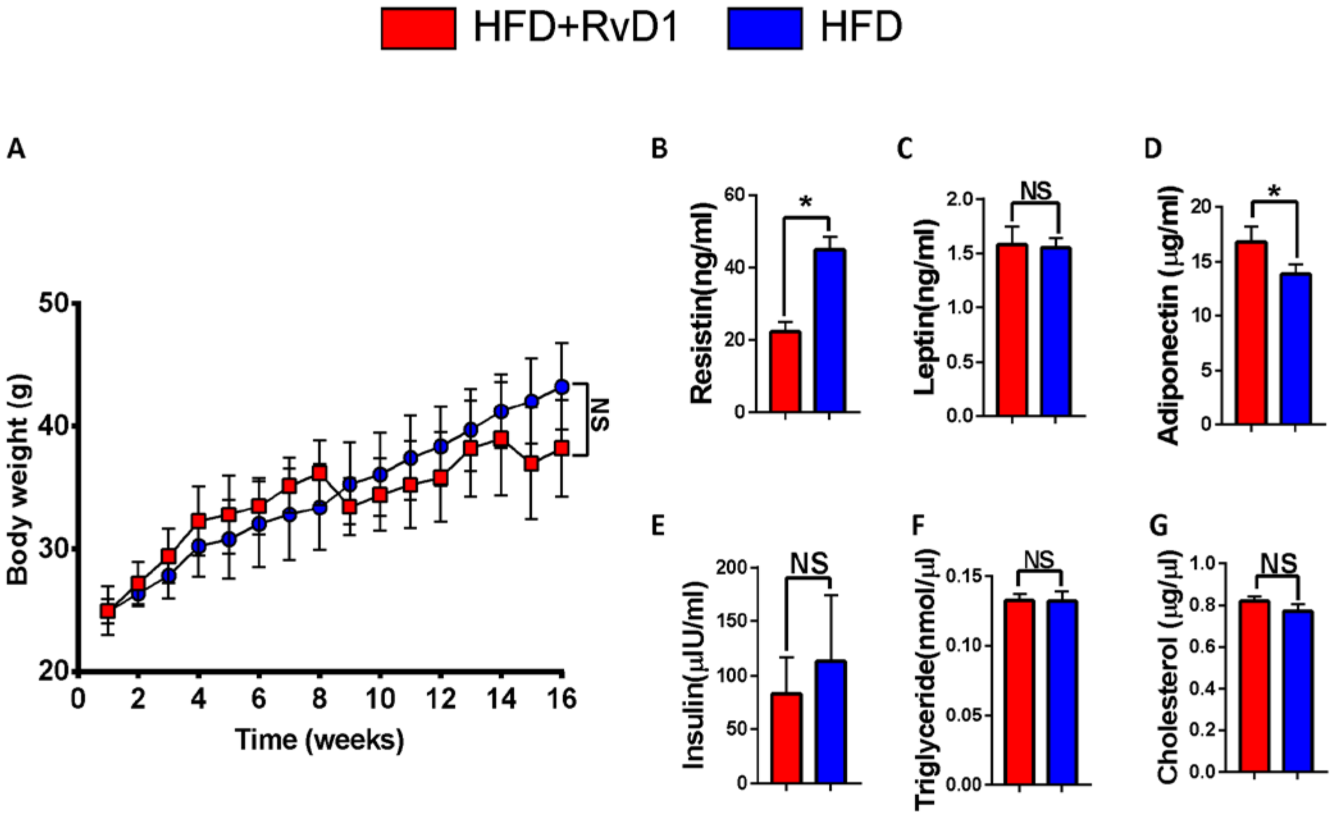

Supplementary Figure 3 Legend: Local treatment of resolvin D1 does not affect bodyweight but altered metabolic parameters. (A) Body weight of HFD+RvD1 or HFD mice were monitored over 16 weeks. (B-J) Effect of RvD1 on metabolic parameters. Measurement of serum resistin (B), leptin (C), adiponectin (D), insulin (E), triglyceride (G) and total cholesterol (H). Graphs represent mean ± SD (n=5). *=p＜0.05. HFD, High-fat diet-fed mice; RvD1, resolvin D1.
